# Supplementary material for: IDO1 Protein Is Expressed in Diagnostic Biopsies from Both Follicular and Transformed Follicular Patients
Source: Int J Mol Sci. 2023 Apr 15;24(8):7314. doi: 10.3390/ijms24087314 (PMC10139172; doi:10.3390/ijms24087314)
Supplement: Supplementary file 1 [file ijms-24-07314-s001.zip › ijms-2304851-supplementary.pdf]

Appendix: Supplementary methods

S1: Patient samples

Immunohistochemical evaluation was performed on pre-therapeutic formalin-fixed, paraffin embedded (FFPE) tumor tissue specimens from a cohort of 53 FL patients diagnosed with a primary diagnosis of FL grade 1-3A at Aarhus University Hospital, Denmark, between 1990 and 2015. This cohort have been described previously[1]. These included 33 patients who had no record of transformation with at least 10 years of follow-up (non-transforming FL, nt-FL) and 20 patients with subsequent histologically confirmed transformation to DLBCL or FL grade 3B, at least 6 months after the primary FL diagnosis (subsequentially-transforming FL, st-FL). In addition, for the 20 st-FL patients, paired transformed lymphoma samples from the time of histological transformation (HT) were also analyzed (histologically transformed FL, tFL)[1]. All biopsies were reviewed by two experienced hematopathologists (SJHD and TLP) and classified according to the 2017 update on the WHO Classification of Tumours of the Haematopoietic and Lymphoid Tissues[2]. Clinicopathological data on all patients were obtained from the Danish Lymphoma Registry[3]. Both clinicopathological and immunohistochemical data on other putative biological markers in this cohort have been published previously[1,4-7]. The study was approved by the Danish National Committee on Health Research Ethics (1-10-72-276-13) and the Danish Data Protection Agency (1-16-02-407-13) and was conducted in accordance with the Declaration of Helsinki.

S2: Immunohistochemical staining for IDO1

Immunohistochemical staining was performed on 4μm FFPE sections using the Ventana Benchmark Ultra automated staining system (Ventana Medical Systems, Tucson, Arizona, USA) using standard methods. Slides were deparaffinized followed by blocking of endogenous peroxidase activity using the OptiView DAB IHC Detection Kit (Ventana, 760-700)[1]. Heat induced epitope retrieval was applied by heating slides to 100ºC for 32 minutes in ULTRA Cell Conditioning Solution 1 (Ventana, 950-224). Primary polyclonal anti-human anti-IDO1 primary antibody (HPA023149, Sigma-Aldrich, Missouri, USA) was diluted 1:100 in Tris buffered antibody diluent (pH 7.2, 15mmol/L NaN3 and stabilizing protein, Dako, Santa Clara, California) followed by 32 minutes of incubation at 37°C. Visualization was performed using the OptiView DAB IHC Detection Kit (Ventana, 760-700) with nuclear counterstaining by hematoxylin. Sections of appendix, tonsil, liver, and pancreas were included on all slides as positive and negative controls[1,4].

S3: Digital image analysis

Stained slides were scanned at a magnification of x20 using the Hamamatsu Nanozoomer 2.0HT scanner (Hamamatsu, Shizouka, Japan). Expression levels of IDO1 were quantified using Visiopharm Integrator system (Visiopharm A/S, Hoersholm, Denmark)[1]. As previously described[1,4], areas of lymphoid tissue suitable for staining quantification were defined by manual outlining of regions of interest (ROI) on each digitized section. Distinct areas of non-lymphoid tissue and technical artefacts were excluded. An analysis protocol package (APP) was designed to quantify the expression levels. Staining quantification outputs were area fractions (AFs), defined as the stained area normalized to the total area within the ROI. Expression levels were calculated on AFs of all positive staining[1,4].

S4: Statistical analysis

Differences in clinicopathological features were assessed using a chi-squared test and Fisher's exact test. Correlation of IDO1 expression and clinicopathological features was evaluated using a Spearman's rank test. Differences in AFs from nt-FL, st-FL, and tFL samples were assessed using an independent Mann-Whitney U test and a paired Wilcoxon ranked sum test. Time-related endpoints were analyzed using the Kaplan Meier and log rank method with overall survival (OS), progression-free survival (PFS), and

transformation-free survival (TFS) as endpoints. OS was defined as time from initial FL diagnosis to the date of death by any cause or censoring. PFS was defined as time from initial FL diagnosis to the date of progression, relapse, HT, death, or censoring. TFS was defined as time from initial FL diagnosis to the date of biopsy-proven HT or censoring[1,4]. Cutoff values for high versus low IDO1 expression for OS, PFS and TFS analyses were determined by a ROC analysis with the optimal cutoff point calculated using Youden's index. P-values below 0.05 were considered statistically significant. Statistical analyses were performed using R Statistical Software (version 4.1.0).

Supplementary figures

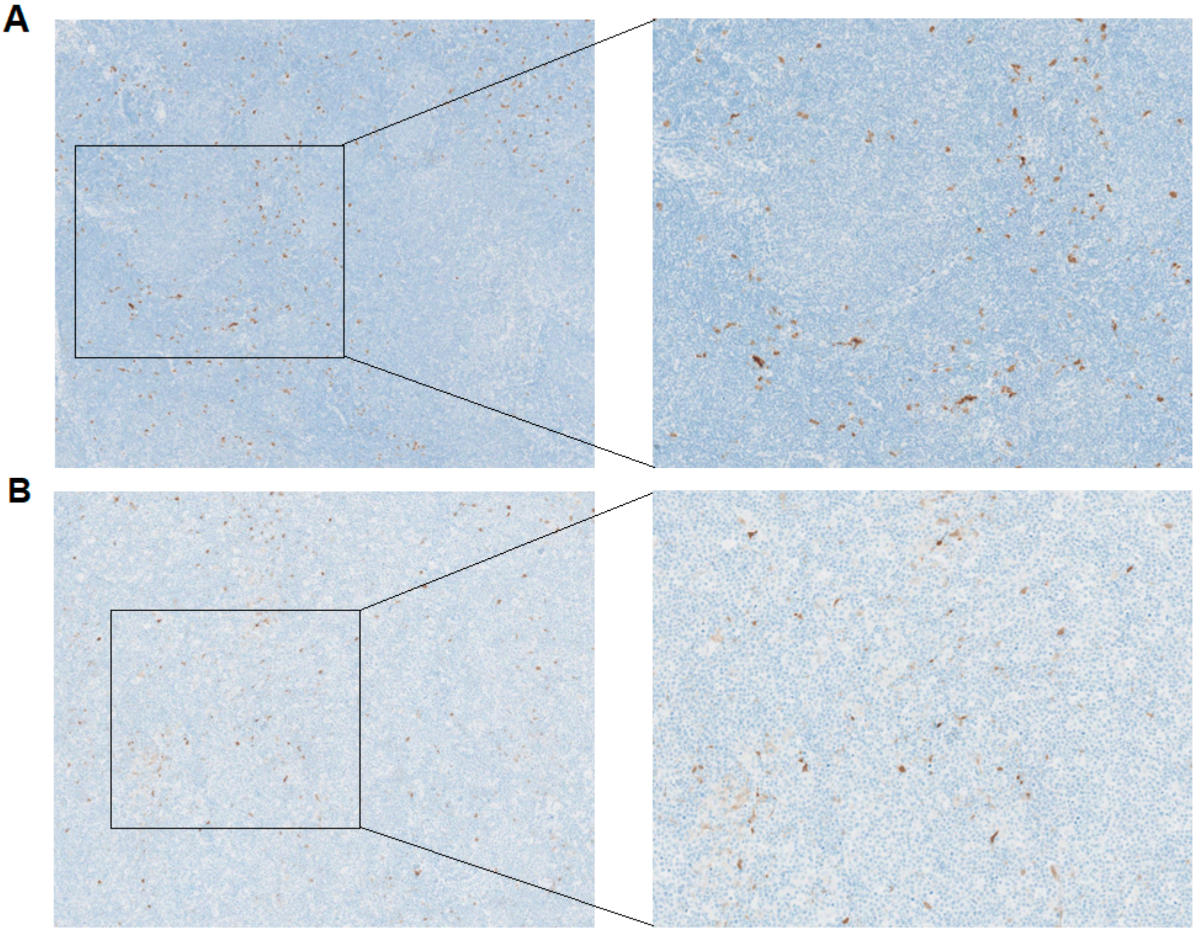

Supplemental Figure S1. Intratumoral expression of IDO1 in st-FL and tFL.
(A) Representative images of immunohistochemical staining of IDO1 in diagnostic st-FL biopsies. Left: magnification of x5, right: magnification of x10. (B) Representative images of immunohistochemical staining of IDO1 in tFL biopsies. Left: magnification of x5, right: magnification of x10.

Supplementary references

1. Enemark MB, Hybel TE, Madsen C, et al. Tumor-Tissue Expression of the Hyaluronic Acid Receptor RHAMM Predicts Histological Transformation in Follicular Lymphoma Patients. Cancers (Basel). 2022;14(5).

2. Swerdlow SH, Campo E, Pileri SA, et al. The 2016 revision of the World Health Organization classification of lymphoid neoplasms. Blood. 2016;127(20):2375-2390.

3. Arboe B, El-Galaly TC, Clausen MR, et al. The Danish National Lymphoma Registry: Coverage and Data Quality. PLoS One. 2016;11(6):e0157999.

4. Beck Enemark M, Monrad I, Madsen C, et al. PD-1 Expression in Pre-Treatment Follicular Lymphoma Predicts the Risk of Subsequent High-Grade Transformation. Onco Targets Ther. 2021;14:481-489.

5. Madsen C, Lauridsen KL, Plesner TL, et al. High intratumoral expression of vimentin predicts histological transformation in patients with follicular lymphoma. Blood Cancer J. 2019;9(4):35.

6. Madsen C, Plesner TL, Bentzen HH, et al. Real world data on histological transformation in patients with follicular lymphoma: incidence, clinico-pathological risk factors and outcome in a nationwide Danish cohort. Leuk Lymphoma. 2020;61(11):2584-2594.

7. Monrad I, Madsen C, Lauridsen KL, et al. Glycolytic biomarkers predict transformation in patients with follicular lymphoma. PLoS One. 2020;15(5):e0233449.
